# Native ion mobility‐mass spectrometry reveals the binding mechanisms of anti‐amyloid therapeutic antibodies

**DOI:** 10.1002/pro.5008

**Published:** 2024-05-09

**Authors:** Yilin Han, Alec A. Desai, Jennifer M. Zupancic, Matthew D. Smith, Peter M. Tessier, Brandon T. Ruotolo

**Affiliations:** ^1^ Department of Chemistry University of Michigan Ann Arbor Michigan USA; ^2^ Department of Chemical Engineering University of Michigan Ann Arbor Michigan USA; ^3^ Biointerfaces Institute University of Michigan Ann Arbor Michigan USA; ^4^ Department of Pharmaceutical Sciences University of Michigan Ann Arbor Michigan USA; ^5^ Department of Biomedical Engineering University of Michigan Ann Arbor Michigan USA

**Keywords:** collision induced unfolding, ion mobility, monoclonal antibody, native mass spectrometry

## Abstract

One of the most important attributes of anti‐amyloid antibodies is their selective binding to oligomeric and amyloid aggregates. However, current methods of examining the binding specificities of anti‐amyloid β (Aβ) antibodies have limited ability to differentiate between complexes that form between antibodies and monomeric or oligomeric Aβ species during the dynamic Aβ aggregation process. Here, we present a high‐resolution native ion‐mobility mass spectrometry (nIM‐MS) method to investigate complexes formed between a variety of Aβ oligomers and three Aβ‐specific IgGs, namely two antibodies with relatively high conformational specificity (aducanumab and A34) and one antibody with low conformational specificity (crenezumab). We found that crenezumab primarily binds Aβ monomers, while aducanumab preferentially binds Aβ monomers and dimers and A34 preferentially binds Aβ dimers, trimers, and tetrameters. Through collision induced unfolding (CIU) analysis, our data indicate that antibody stability is increased upon Aβ binding and, surprisingly, this stabilization involves the Fc region. Together, we conclude that nIM‐MS and CIU enable the identification of Aβ antibody binding stoichiometries and provide important details regarding antibody binding mechanisms.

## 1 INTRODUCTION

Alzheimer's disease (AD) is a neurodegenerative disease that affects over 6 million people in the U.S. (2019 Alzheimer's Disease Facts and Figures, 2019). AD pathology is complicated and several compelling hypotheses have been developed (Markesbery, 1997; Arnsten et al., 2021; Hardy & Allsop, 1991), with the most prominent being the amyloid hypothesis centering on the role of amyloid‐β (Aβ) peptides (John & Gerald, 1992). As such, Aβ has long been one of the most important targets for AD drug development. Biotherapeutic monoclonal antibodies (mAbs) have been enormously successful in recent years in the treatment of myriad diseases, including cancer, arthritis, and autoimmune disorders (Lu et al., 2020). For example, a recent report indicated that mAbs represented 5 of the top 10 bestselling drugs in 2021 (Urquhart, 2022). Compared to small molecule drugs, mAbs tend to exhibit lower risks of off‐target effects and offer less frequent dosing options for patients (Rabia et al., 2018; Tiller & Tessier, 2015).

Recently, Aβ has been successfully targeted clinically by multiple mAbs for the first time. Aducanumab was the first mAb to be approved by the FDA, and it has been reported to possess conformational selectivity for various types of Aβ aggregates (Arndt et al., 2018; Linse et al., 2020; Soderberg et al., 2023), including small oligomers theorized to be cytotoxic and involved in AD etiology (FDA Grants Accelerated Approval for Alzheimer's Drug | FDA, 2022). Likewise, a second Aβ antibody (lecanemab) was also recently approved for treating AD, and this antibody also displays conformational specificity for Aβ aggregates (Soderberg et al., 2023). These approvals are potentially exciting since they are the first therapeutics to target an underlying cause of AD and follow a long list of failed AD drug candidates (Mullard, 2019). However, there are many challenges remaining in this space. For example, aducanumab had a difficult road to approval and there remain many questions surrounding its potential efficacy (Knopman et al., 2021; Mullard, 2021). One of the important critical quality attributes (CQAs) evaluated during mAb development relates to its selective binding to intended targets. However, for mAbs targeting Aβ aggregates, the current methods of examining binding specificity usually involve immunoprecipitation or gel pull‐down assays, and these methods typically lack the resolution to differentiate between different mAb‐Aβ complexes that may form (Meilandt et al., 2019; Rofo et al., 2021). Quite often, the specific oligomeric Aβ species bound to potential mAb therapeutics under development are not evaluated in detail. As such, the development of new methods capable of evaluating mAb‐Aβ complexes at high resolution is critical for defining antigen specificity and associated CQAs mAb‐based AD drug candidates targeting such oligomeric species.

Here, we develop a native ion mobility mass spectrometry (nIM‐MS) method for the acquisition of enhanced information content for mAb‐oligomer binding assays, while simultaneously reducing the typical assay cost in terms of the material used and time involved. Generally, nIM‐MS is a multidimensional technology that allows rapid protein structure, mass, and stability analysis. The nIM‐MS technique employs nano‐electrospray (nESI) conditions designed to preserve non‐covalent protein‐ligand complexes without the need for crosslinking (Erba & Petosa, 2015). IM separates ions according to differences in ion collision cross section (CCS) and charge. An extension of IM‐MS, collision induced unfolding (CIU) experiments activate and unfold proteins by increasing collision energy prior to IM separation, allowing measurement of gas‐phase stability and unfolding patterns (Dixit et al., 2018). Together, nIM‐MS has demonstrated utility for the study of mAb higher order structure (HOS) related to the evaluation of mAb sequences (Watanabe et al., 2018; Hernandez‐Alba et al., 2018), disulfide bonding patterns (Bagal et al., 2010), glycosylation (Tian & Ruotolo, 2018; Tian et al., 2015) and is able to differentiate between biosimilars and innovator mAbs (Vallejo et al., 2021).

In this report, we aim to address the gaps in standard methods for the evaluation of mAb binding specificity to Aβ oligomers. We describe a set of nIM‐MS measurements acquired across three mAbs: aducanumab, crenezumab (a mAb with low conformational specificity for Aβ that failed clinical AD trials in 2019) (Meilandt et al., 2019; Guthrie et al., 2020; Genentech, 2019), and A34 (a mAb produced through affinity maturation for improved sequence and conformational specificity targeting Aβ fibrils) (Desai et al., 2021). We evaluate these mAbs against their common antigen, Aβ, across three different Aβ proteoforms and a range of in vitro conditions designed to promote different amounts of Aβ oligomers. Our data indicate clear evidence of Aβ binding to all three mAbs resulting in diverse binding stoichiometries ranging from 1:1 to 1:4 mAb:Aβ complexes. We also observed differences in binding between mAbs associated with their expected conformational or sequence specificities, detecting strong evidence of oligomer binding for only A34. Our CIU analyses detect a stabilizing effect upon mAb:Aβ complex formation, with different CIU signatures associated with increases in complex stability measured upon Aβ attachment for each of the mAbs tested. Our method is further validated through negative control data indicating the binding data collected during our experiments is not a result of nESI related artifacts. We conclude by discussing how nIM‐MS can be used for future mAb discovery and development efforts that aim to target cytotoxic Aβ species as well as similar polydisperse oligomeric peptides and proteins.

## 2 RESULTS AND DISCUSSION

**FIGURE 1:**
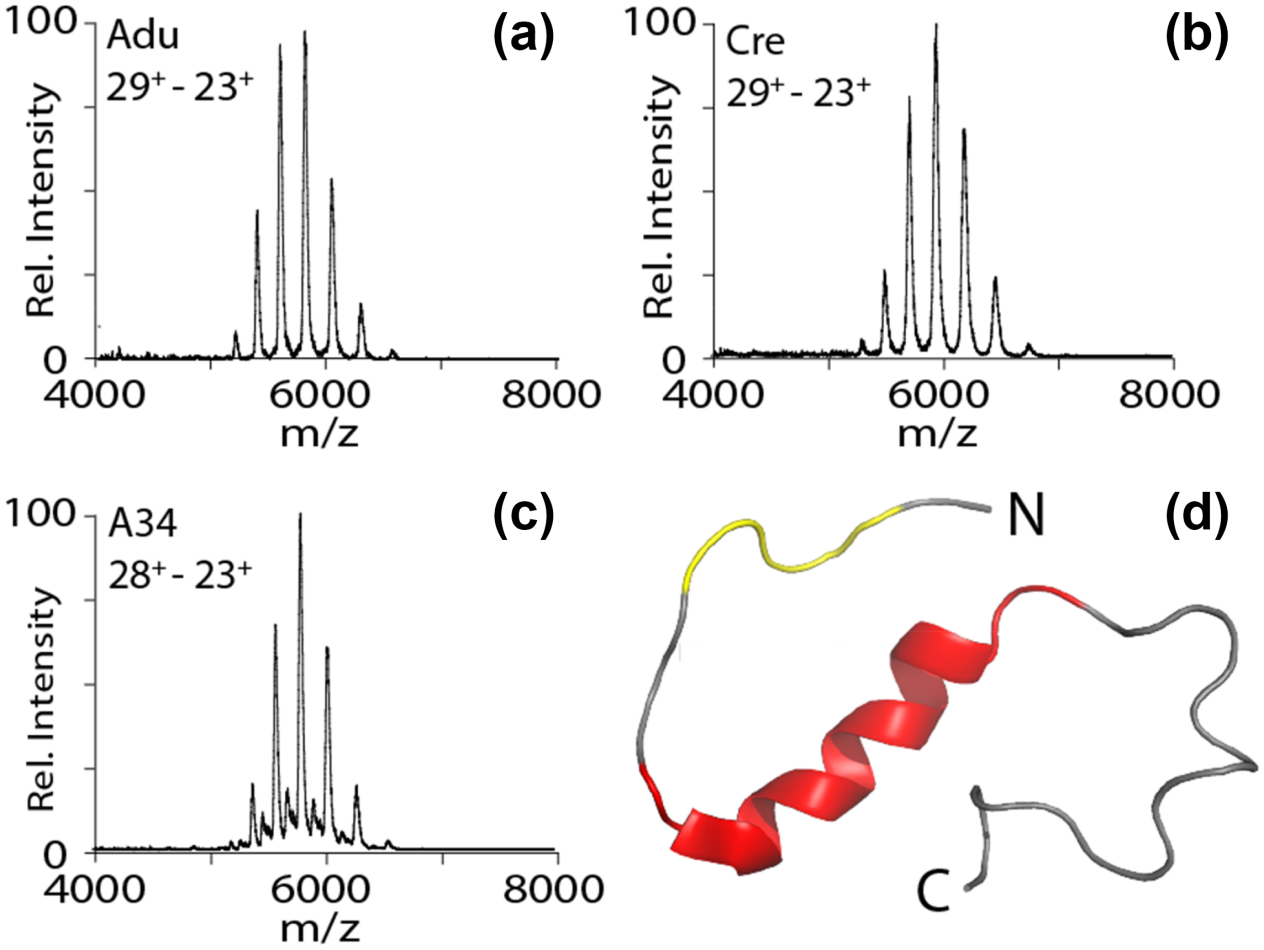
Mass spectra of Adu (a), Cre (b), and A34 (c) in native conditions. NMR structure (d) of Aβ_40_ monomer (PDB: 2LFM) with the reported epitope of both Adu and A34 highlighted in yellow and the reported epitope of Cre highlighted in red.

Three mAbs were chosen for our experiments to evaluate the ability of nIM‐MS to detect specific non‐covalent complexes formed with Aβ monomers and oligomers based on their conformational binding specificity (Desai et al., 2021). Figure 1 contains mass spectra recorded for these three mAbs under native conditions, revealing monomer charge states ranging from 23+ to 29+ for Adu (Figure 1a) and Cre (Figure 1b). Deconvolution of the mass spectra placed the MW of Adu at 150 kDa and Cre at 148 kDa. Adu and Cre were expressed with their variable regions grafted into a common IgG1 framework, thus producing minor MW differences between the mAbs we evaluated here and the clinical‐stage mAbs reported on previously (FDA, 2021; ChemIDplus ‐ 1095207‐05‐8, 2022). MS data recorded for A34 (Figure 1c) contained monomer charge states ranging from 23+ to 28+ and a molecular mass of 150 kDa for the major component detected. Satellite signals can be observed between the main peaks detected in Figure 1c indicating the presence of a 147 kDa truncated form of A34 produced during the antibody production process. The binding epitope of the two commercial antibodies has been previously reported (Vallejo et al., 2021; Ultsch et al., 2016) and is highlighted on the NMR structure of the Aβ monomer shown (Figure 1d). Adu recognizes Aβ residues 3–7 for binding (Vallejo et al., 2021), while Cre recognizes Aβ residues 11–25 (Ultsch et al., 2016) and A34 recognizes an epitope on the N‐terminus of Aβ similar to that utilized by Adu (Tian et al., 2015).

**FIGURE 2:**
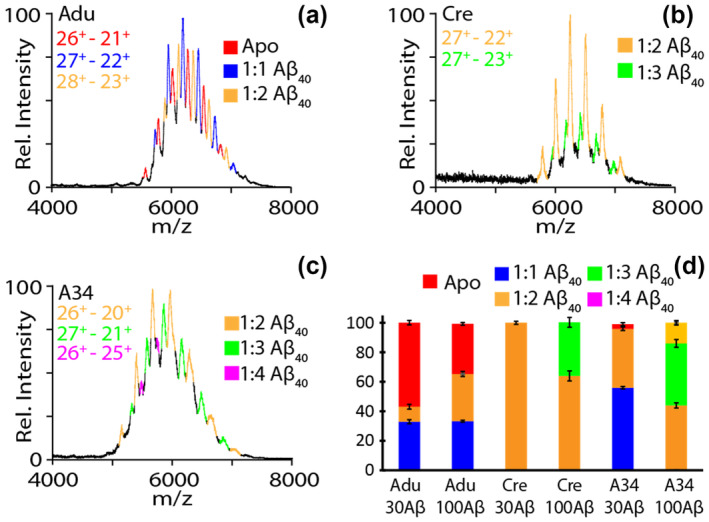
Mass spectra recorded for Adu (a), Cre (b), and A34 (c) in the presence of 100 μM Aβ40 in solution. The observed binding stoichiometries and charge states are labeled in corresponding colors indicated in the key shown. A stacked bar plot (d) of the %antibody bound for Adu, Cre, and A34.

When incubated with 10× (30 μM) and 33× (100 μM) excesses Aβ_40_, mAb:Aβ complexes can be directly observed using nIM‐MS for Adu, Cre and A34. The two concentrations of Aβ_40_ are used in our experiments in order to generate a range of Aβ oligomer forms, ranging from dimers to pentamers (Figure S1a). For samples containing 10× Aβ_40_, only Aβ monomers, dimers and trimers were detected free in solution, with Adu and A34 exhibiting 1:1 and 1:2 mAb:Aβ binding stoichiometries (Figure S2a,b). Interestingly, Cre nIM‐MS data exclusively contained signals corresponding to a 1:2 mAb:Aβ stoichiometry (Figure S2c). This exclusivity aligned well with the low conformational specificity previously reported for Cre, likely corresponding to a single Aβ_40_ monomer bound to each Fab. At 33× excess Aβ_40_, we observe a significant increase in the amount of trimeric and tetrameric Aβ_40_ species free in solution (Figure S1b). While Cre and Adu appear to possess similar Aβ complex stoichiometries under these conditions when compared to data recorded for less concentrated Aβ samples, nIM‐MS data recorded for A34 reveals evidence of 1:3 and 1:4 mAb:Aβ stoichiometries (Figure 2a–c). Furthermore, it is worth noting that given their similar reported conformational specificities, Adu and A34 display significantly different apparent Aβ binding affinities, with apo A34 representing only 3.1% of the recorded signal intensity when incubated with 10× Aβ_40_ samples, while the same feature for Adu accounts for 56% of the signal intensity recorded under the same conditions (Figure 2d).

**FIGURE 3:**
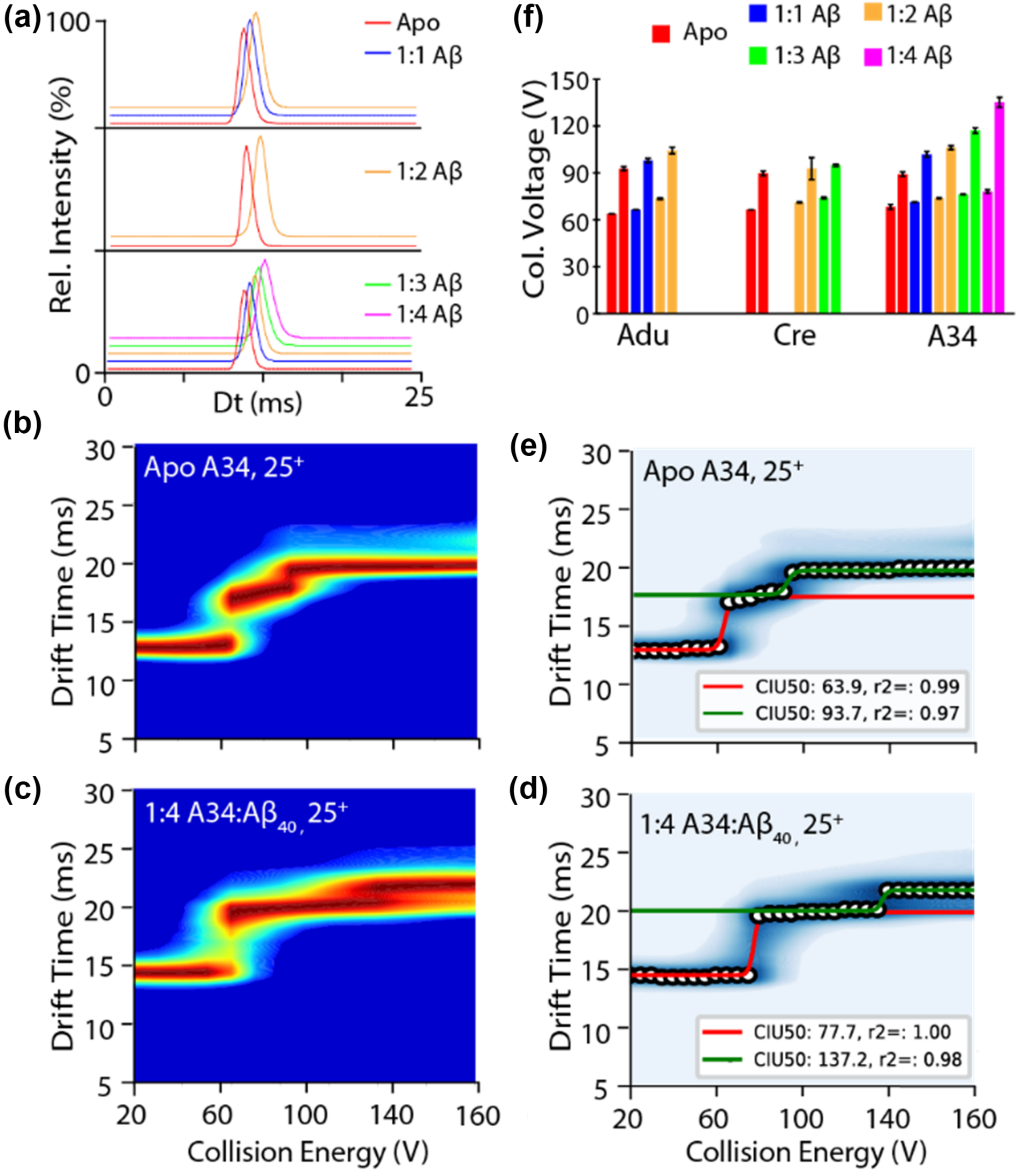
Arrival time distribution plots of 25+ apo and bound states of Adu, Cre, and 97A34 with 100 μM Aβ_40_ present in solution (a). CIU fingerprints of the 25+ charge state of apo 97A34 (b) and 1:4 bound 97A34:Aβ (c). Identified CIU features and CIU 50 values are calculated in (d) for 1:4 antibody:Aβ bound complex and (e) for apo 97A34. In panels (e) and (d), sigmoid fits are shown for CIU50‐1 (red) and CIU50‐2 (green) measurements, with corresponding CIU50 values and correlation coefficients for the fits indicated in the associated legends. Bar plot (f) of the CIU50 values of all three antibodies with all binding stoichiometries observed.

**FIGURE 4:**
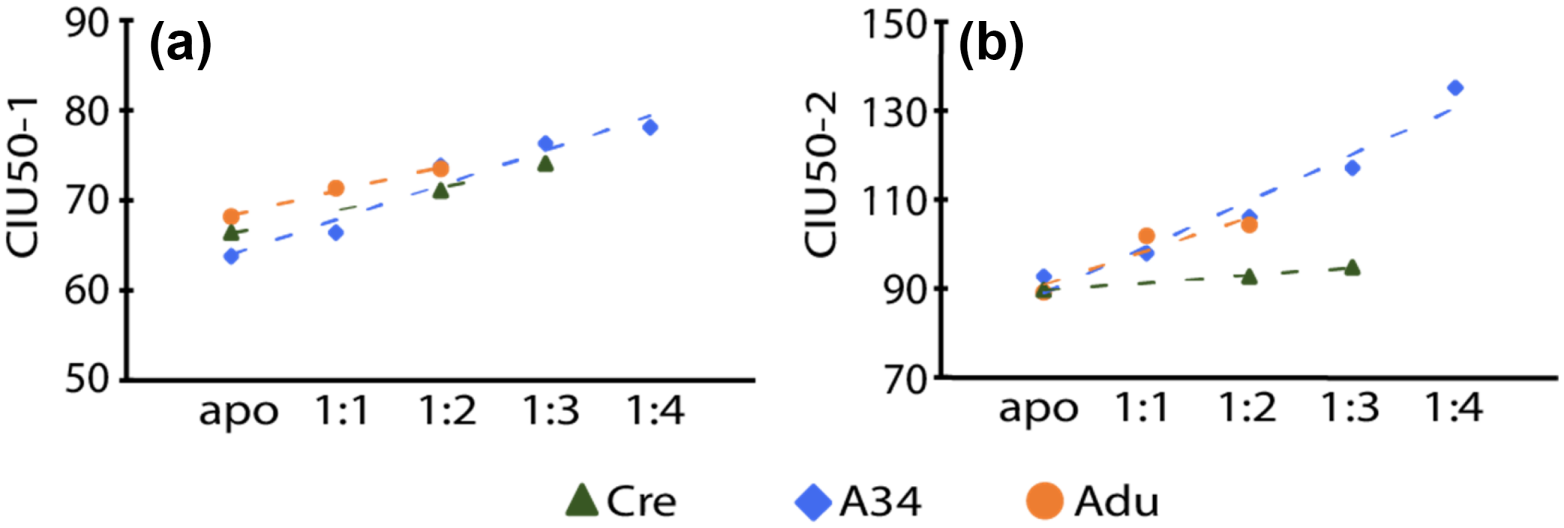
CIU stability trend data recorded for Adu, Cre and 97A34 using CIU50‐1 (a) and CIU50‐2 (b) values. Trendlines shown represent linear fits to the data. Details of the fit for (a) Adu: slope = 2.65, *R* ^2^ = 0.99, Cre: slope = 2.53, *R* ^2^ = 0.99, and A34: slope = 3.85, *R* ^2^ = 0.95. (b) Adu: slope = 7.6, *R* ^2^ = 0.95, Cre: slope = 1.7, *R* ^2^ = 0.99, and A34: slope = 10.4, *R* ^2^ = 0.95.

Though our nIM‐MS data produced clear evidence of mAb:Aβ_40_ complexes, any changes to mAb HOS upon Aβ binding was not readily detected in this mode of operation. For example, Figure 3a displays the IM arrival time distributions (ATDs) for all mAb:Aβ_40_ complexes observed in our experiments. Overall, we observe shifts in the recorded centroid ATDs (and by extension, the CCS) for mAb complexes in a manner correlated with the number of Aβ_40_ bound, an effect that we attribute to the associated increases in molecular weight produced upon Aβ binding and not to any associated HOS changes in the mAb. To evaluate mAb:Aβ_40_ complex HOS in more detail, we recorded CIU data for mAb:Aβ_40_ complexes. Our experiments evaluated a wide range of mAb‐Aβ complex charge states, and for the CIU analyses shown here we focused on 25+ ions, as they exhibited superior reproducibility and were consistently observed across all mAb samples incubated at both Aβ_40_ concentrations tested. For example, we observed three features in both our apo and our 1:4 A34:Aβ_40_ complex CIU data (Figure 3b,c). CIU fingerprint data recorded for Adu and Cre samples produced similar features to those shown in Figure 3 (Figure S3). By fitting sigmoid curves to the CIU data, we are able to extract CIU50 values which correspond to the accelerating potential necessary to convert 50% of the preceding CIU feature into the following state detected in the assay. Such values have been observed previously to report on domain‐specific stability values within the detected mAb:Aβ complexes (Villafuerte‐Vega et al., 2023). Analysis of this data reveals CIU50‐1 values of 63.9 ± 0.6 and 77.7 ± 1.2 as well as CIU50‐2 values of 97.7 ± 1.2 and 137.2 ± 3.2 for apo and 1:4 A34:Aβ_40_ complexes respectively, indicating an increase in mAb stability upon binding (Figure 4d,e) Such increases in stability are also observed for Adu and Cre‐Aβ complexes, although the extent of stabilization observed upon Aβ binding appears to be uniquely related to the mAb tested, with Adu and A34 increasing in stability to a greater extent than Cre upon Aβ_40_ attachment when compared across identical complex stoichiometries (Figure 3f).

A more detailed analysis of our CIU‐50 values (Figure 4) reveals different trends associated with the stability increases observed in mAb samples upon Aβ_40_ binding. For CIU50‐1 values, we observe relatively tight correlations between the stability shifts observed as a function of peptides bound to all three mAbs studied here (Figure 4a). In contrast, CIU50‐2 values reflect the greater enhancement in mAb stability upon Aβ_40_ binding for Adu and A34 referenced above (Figure 4b). Prior work has indicated that CIU50‐1 and CIU50‐2 values are related to Fab and Fc unfolding processes respectively, indicating non‐local HOS involvement in Adu and A34 Aβ_40_ binding (Villafuerte‐Vega et al., 2023). The nIM‐MS data described above has been focused on Aβ_40_, the most abundant Aβ proteoform present in vivo (Spies et al., 2010). However, in certain forms of AD, the concentration of Aβ_42_ increases dramatically, and has been observed to be more aggregation‐prone and more toxic than Aβ_40_ (Irvine et al., 2008). Overall, over 26 Aβ proteoforms have been identified in the human brain, further emphasizing the need for methods such as nIM‐MS for evaluating complex proteoform‐specific oligomer targeting (Wildburger et al., 2017).

**FIGURE 5:**
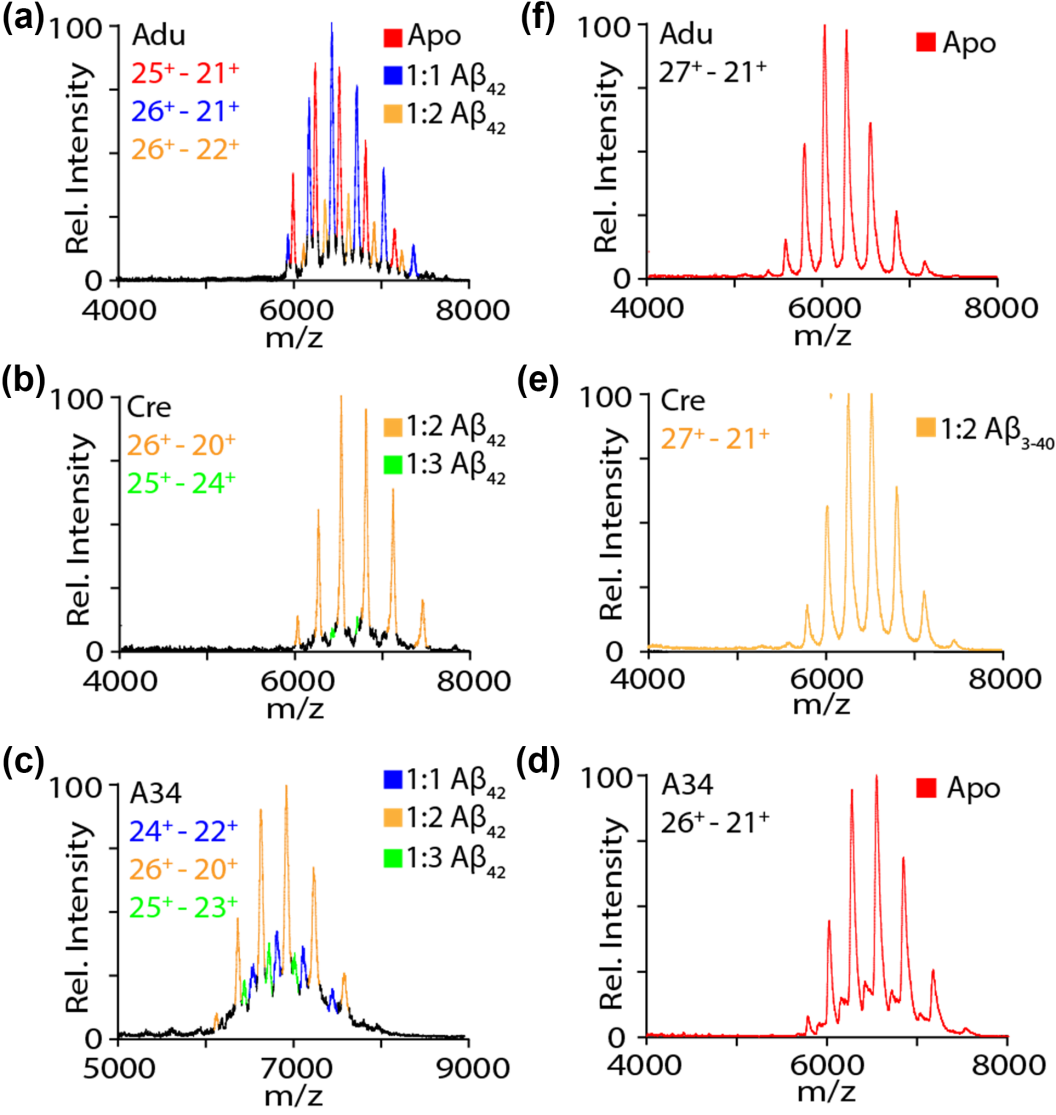
Mass spectra of Adu (a), Cre (b), and 97A34 (c) with 30 μM Aꞵ_42_ or Aꞵ_3‐40_ incubated with A34 (d), Cre (e) and Adu (f) respectively.

To pursue such objectives, we repeated the nIM‐MS binding experiments described above using both Aβ_42_ and Aβ_3‐40_, with the latter proteoform included, in part, to test the fidelity of nIM‐MS data recorded for Adu and A34, both of which rely on an N‐terminal epitope for Aβ binding. Overall, our data (Figure 5) reveals similar binding trends for Aβ_42_ as observed for Aβ_40_, with stoichiometries of both 1:1 and 1:2, exclusively 1:2, and a mixture of 1:1, 1:2 and 1:3 observed for Adu, Cre and A34 Aβ_42_ complexes respectively. Overall, our Aβ_42_ indicates a lesser amount of attached monomer and oligomer to all the mAbs studied here when compared to our Aβ_40_ data shown in Figure 2, which is likely a reflection of the more rapid aggregation of Aβ_42_, which leads to less available monomer and oligomer available in solution for mAb binding. In addition, our Aβ_3‐40_ data confirms the absence of complexes for Adu and A34, but retained detection of 1:2 Cre:Aβ complexes, as expected. Importantly, our Aβ_3‐40_ results do not vary significantly at 100 mM Aβ_3‐40_ concentrations, ruling out any significant influences of nESI artifacts in our nIM‐MS mAb:Aβ binding data (Tamara et al., 2022).

**FIGURE 6:**
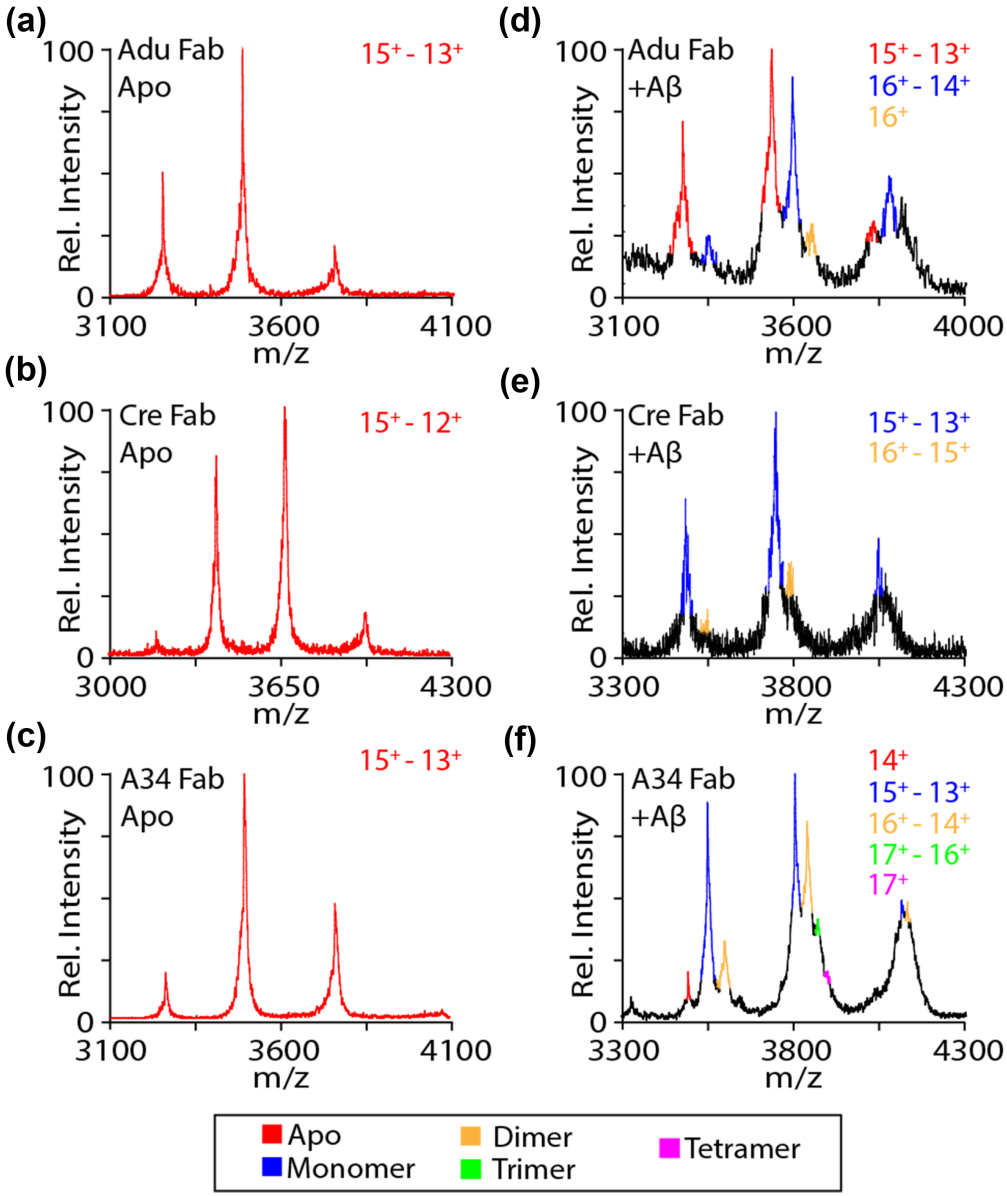
Mass spectra of apo Fab from Adu (a), Cre (b) and A34 (c). Mass spectra of Fab Adu (d), Cre (e) and A34 (f) incubated with 100 μM Aꞵ_40_.

While our nIM‐MS data for full‐length mAbs reveals higher‐stoichiometry complexes that may be indicative of oligomeric Aβ binding in some cases, given the presence of two independent Fab binding locations on each mAb, interpreting the significance of 1:2 and higher‐order mAb:Aβ complexes detected in the context of such Aβ oligomer targeting is challenging. In order to clarify and confidently assign our full‐length mAb results, we produced individual Fab arms of each of the mAbs measured in the experiments described above and incubated these samples with 100 μM Aβ_40_ under native conditions. For the nIM‐MS data shown in Figure 6, any Fab:Aβ complex stoichiometries detected beyond 1:1 can be treated as direct evidence of Fab oligomer binding. The deconvoluted molecular mass values recorded for each Fab were 48.78, 47.57 and 48.9 kDa, for Adu, Cre and A34 respectively, all of which conform to sequence mass expectations. For both Adu and Cre (Figure 6d,e), small signals for 1:2 complexes are detected, indicating limited oligomer binding capacity. In contrast, more significant signals for 1:2 and 1:3 Fab:Aβ complexes were detected for A34 (Figure 6f), indicating a stronger preference for Aβ_40_ oligomers for A34 antibody in comparison to the other antibodies tested in this study.

## 3 CONCLUSIONS

Here we present an information‐rich nIM‐MS method for probing mAb binding specificity within polydisperse oligomeric populations of Aβ proteoforms. Our nIM‐MS data were able to distinguish the different binding behaviors in Adu, Cre and A34 mAbs when challenged with different populations of Aβ oligomers. Specifically, we demonstrate evidence of significant Aβ oligomer binding for A34, and primarily Aβ monomer binding for the other two mAbs tested, despite expected oligomer engagement for Adu. Our oligomer assignments are supported by the MS data acquired for purified mAb and Aβ reference samples, as well as Fab fragment Aβ binding data, which allows us to confidently discern the presence or absence of mAb‐bound Aβ oligomers. Furthermore, we find that CIU provides unique insights into the operative mechanisms associated with mAb‐Aβ binding. When analyzed in detail, our data indicates that the Aβ binding stoichiometry‐dependent stability enhancements observed in conformational mAbs is driven, in part, through Fc domain involvement, a mAb region remote from the intended binding domain. Conversely, the non‐conformational Aβ binding events observed for Cre appear to produce smaller stability shifts that are more uniformly distributed between Fab and Fc regions. Future efforts associated with CIU and nIM‐MS method development should focus on investigating the properties and utility of the stability data, such as the data in Figure 5, during the design phase of mAb therapeutics intended to target specific conformational or oligomeric states within polydisperse ensembles. In addition, the typical mAb detection limit for nIM‐MS is in the high nM range, and thus any improvements in ion transmission efficiency would produce correlated improvements in utilizing this approach to assess mAb:antigen binding constants.

While the nIM‐MS and CIU methods discussed here have focused only on mAb:Aβ complexes, our approach should be extendable to most antibody:antigen systems and be especially useful when target antigens exist across an array of conformations or oligomeric states. Our experimental procedures are label‐free, rapid, and require relatively little sample when compared to standard methods commonly used to evaluate mAb binding specificity. Importantly, nIM‐MS enables the direct recording of intact mAb molecular mass, antigen binding, and target oligomerization in parallel with CIU assays in a single experimental frame. Such data can be recorded rapidly (5–30 s for complete nIM‐MS and CIU datasets for an individual sample), paving the way for such experiments to be deployed as an information‐rich screening technology capable of guiding mAb discovery and optimization. Future efforts in our lab will be focused on directly demonstrating the utility of nIM‐MS and CIU screening technologies for the discovery and classification of therapeutic mAbs. In addition, we intend to deploy nIM‐MS technologies to study samples of clinical relevance containing amyloid plaques and biological membranes, thus catalyzing the production of next‐generation biotherapeutics aimed at treating debilitating ailments associated with protein misfolding and aggregation.

## 4 MATERIALS AND METHODS

Aducanumab (Adu), crenezumab (Cre) and A34 were prepared using methods previously described (Guthrie et al., 2020; Desai et al., 2021; Arndt et al., 2018). Purified antibodies were buffer exchanged into 200 mM ammonium acetate (pH 7.4) using Bio‐Rad Bio‐Spin P‐30 columns with a 30 k MWCO. The protein concentration after buffer exchange was assayed using a Thermo Scientific NanoDrop 2000 UV–Vis spectrophotometer (Vernon Hills, IL, USA). Aβ_40_, Aβ_42_, and Aβ_3‐40_ were purchased from Anaspec (Fremont, CA, USA). All Aβ were prepared by dissolving in 30–50 μL 1% [v/v] ammonium hydroxide solution first, then diluted with 200 mM ammonium acetate solution (Sigma‐Aldrich, St. Louis, MO, USA), pH 7.4 to create stock solutions. Peptide concentrations for the stock solutions were calculated from absorbance at 280 nM using a Thermo Scientific NanoDrop 2000 UV–Vis spectrophotometer (Vernon Hills, IL, USA).

The V_L_‐C_L_ and V_H_‐C_H_1 regions of the analyzed aducanumab, crenezumab and A34 antibodies were cloned into mammalian expression plasmids for the production of Fabs. A 6**×**‐His tag was added to the C‐terminus of C_L_ for Fab purification. HEK293‐6E cells were transiently transfected with these plasmids, and Fabs were purified with Ni‐NTA agarose resin after 6 days. Fab concentration was determined by measuring the absorbance at 280 nm using a NanoDrop, and Fab purity was analyzed by SDS‐PAGE and size‐exclusion chromatography.

Aducanumab, crenezumab and A34 were kept at constant concentration of 3 μM while Aβ proteforms prepared at concentrations of either 30 μM (10**×** excess) or 100 μM (33**×** excess). Aβ_40_ and Aβ_3‐40_ samples were first aggregated by shaking and incubating at 37°C for 1 h prior to mixing with mAbs prior to nIM‐MS analysis. Due to the rapid aggregation of Aβ_42_, these samples were not pre‐incubated but instead directly added to the mAb samples prior to nIM‐MS. Fabs of each mAb are incubated with 100 μM Aβ_40_ in a similar manner.

IM‐MS data was collected on a quadrupole ion‐mobility time‐of‐flight (ToF) mass spectrometer (Synapt G2 HDMS, Waters, Milford, MA, USA) with a nano‐electrospray ionization (nESI) source. The source was operated at positive mode with the nESI voltage set at 1.0–1.3 kV, the sampling cone was set to 25–35 V and bias was set to 45 V. The source temperature was set to 20°C. The backing pressure was set to 7–7.5 mbar. The traveling wave IM chamber was operated at a pressure of approximately 3.3 mbar with a traveling wave height and velocity set at 500 m/s and 30 V, respectively. The m/z window was set from 500 to 15,000 m/z with a ToF pressure of 1.6 x 10^−6^ mbar. IM‐MS data were analyzed using MassLynx 4.1 and Driftscope 2.0 software (Waters, Milford, MA, USA). ESIprot was used to deconvolute the mass spectra recorded (Winkler, 2010). Ions were subjected to collisions in the traveling wave ion trap prior to IM separation to perform all charge state CIU experiments. The collision voltage was ramping from 5 to 150 V in increments of 10 V/step. Triplicate experiments were performed. Drift time were extracted at each collision step with TWIMExtract (Haynes et al., 2017). These extracted drift time data were then analyzed using a home‐built software package CIUSuite 2.2 (Polasky et al., 2019). Mass shifts of the mAb:Aβ_40_ complexes are confirmed by an increased MW of 4434 Da for 1:1 complexes, 8.9 kDa for 1:2 complexes, 13 kDa for 1:3 complexes and 17.4 kDa for 1:4 complexes.

## AUTHOR CONTRIBUTIONS

**Brandon T. Ruotolo:** Conceptualization; investigation; funding acquisition; writing – original draft; methodology; writing – review and editing; supervision; resources. **Yilin Han:** Conceptualization; investigation; writing – original draft; methodology; validation; writing – review and editing; data curation. **Alec A. Desai:** Writing – review and editing; resources; methodology; conceptualization; investigation. **Jennifer M. Zupancic:** Conceptualization; investigation; methodology; writing – review and editing. **Matthew D. Smith:** Writing – review and editing. **Peter M. Tessier:** Conceptualization; investigation; funding acquisition; writing – review and editing; methodology; supervision; resources.

## Supporting information


**FIGURE S1.** IM‐MS drift scope data of Adu with 30 μM (a) and 100 μM (b) Aβ_40_ present in solution. Free Aβ_40_ oligomers are identified in ovals.
**FIGURE S2.** Mass spectra recorded for Adu (a), A34 (b), and Cre (c) with 30 μM Aβ_40_ present in solution.
**FIGURE S3.** CIU fingerprint of full length mAbs and the mAbs:Aβ40 complexes at 25+ charge state: (a) Apo Adu, (b) 1:1 Adu:Aβ_40_, (c) 1:2 Adu:Aβ_40_, (d) Apo Cre, (e) 1:2 Cre:Aβ_40_, (f) 1:2 Cre:Aβ_40_, (g) 1:1 A34:Aβ_40_, (h) 1:2 A34:Aβ_40_.
**FIGURE S4.** Pie chart depicting the percentage of bound Aβ40 for Adu, Cre, and A34 at concentrations of 30 and 100 μM.
