# Supplementary material for: Native ion mobility‐mass spectrometry reveals the binding mechanisms of anti‐amyloid therapeutic antibodies
Source: Protein Sci. 2024 May 9;33(6):e5008. doi: 10.1002/pro.5008 (PMC11081520; doi:10.1002/pro.5008)
Supplement: Supplementary file 1 — FIGURE S1. IM‐MS drift scope data of Adu with 30 μM (a) and 100 μM (b) Aβ40 present in solution. Free Aβ40 oligomers are identified in ovals. FIGURE S2. Mass spectra recorded for Adu (a), A34 (b), and Cre (c) with 30 μM Aβ40 present in solution. FIGURE S3. CIU fingerprint of full length mAbs and the mAbs:Aβ40 complexes at 25+ charge state: (a) Apo Adu, (b) 1:1 Adu:Aβ40, (c) 1:2 Adu:Aβ40, (d) Apo Cre, (e) 1:2 Cre:Aβ40, (f) 1:2 Cre:Aβ40, (g) 1:1 A34:Aβ40, (h) 1:2 A34:Aβ40. FIGURE S4. Pie chart depicting the percentage of bound Aβ40 for Adu, Cre, and A34 at concentrations of 30 and 100 μM. [file PRO-33-e5008-s001.docx]

Supplementary Material

Native Ion-Mobility Mass Spectrometry Reveals the Binding Mechanism of Anti-Amyloid Therapeutic Antibodies

Yilin Han^1^, Alec A. Desai^2,3^†, Jennifer M. Zupancic^2,3^, Matthew D. Smith^2,3^, Peter M. Tessier^2,3,4,5^, Brandon T. Ruotolo^1^*

1. Department of Chemistry 2. Department of Chemical Engineering 3. Biointerfaces Institute 4. Department of Pharmaceutical Sciences 5. Department of Biomedical Engineering, University of Michigan, Ann Arbor, 48109, United States

*Brandon T. Ruotolo Email: [bruotolo@umich.edu](mailto:bruotolo@umich.edu)


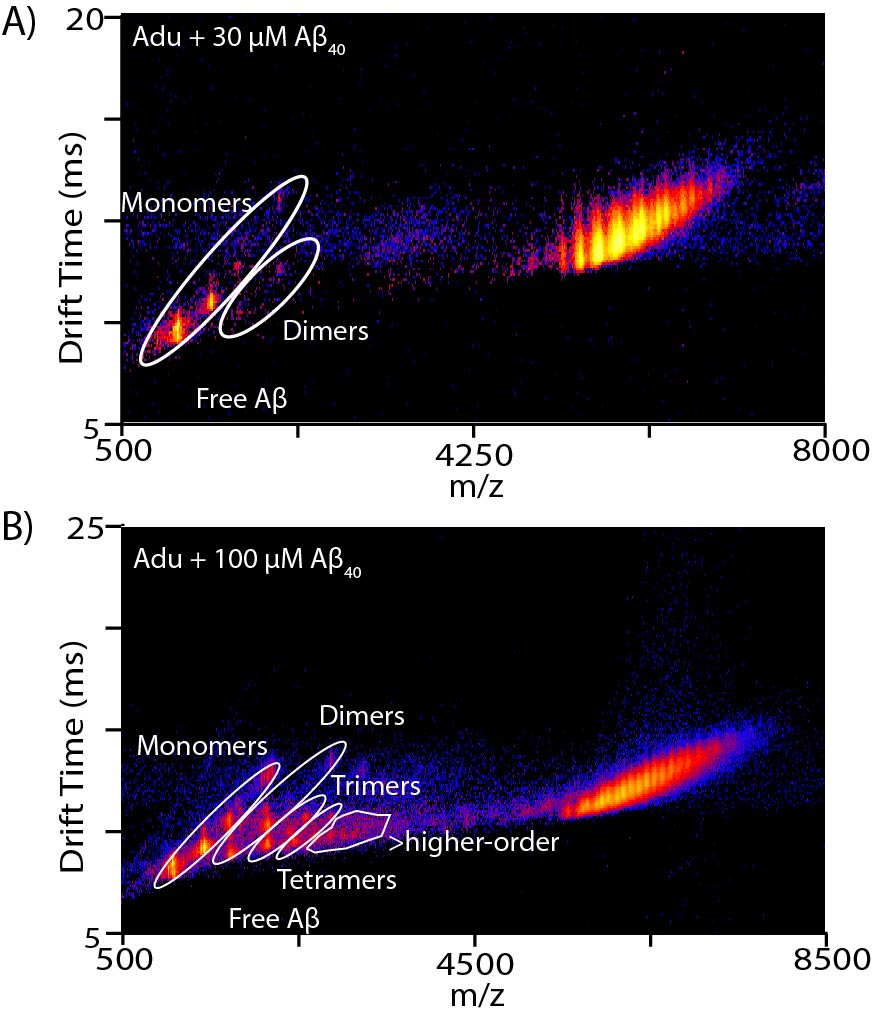


Figure S1 IM-MS drift scope data of Adu with 30 µM (A) and 100 µM (B) Aꞵ_40_ present in solution. Free Aꞵ_40_ oligomers are identified in ovals.


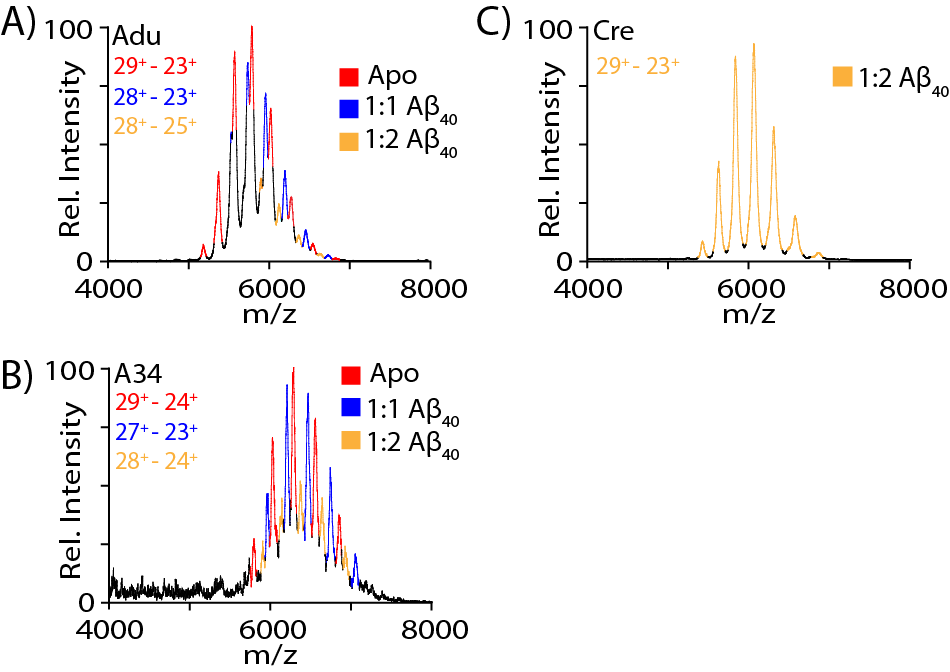


Figure S2 Mass spectra recorded for Adu (A), A34 (B), and Cre (C) with 30 µM Aꞵ_40_ present in solution


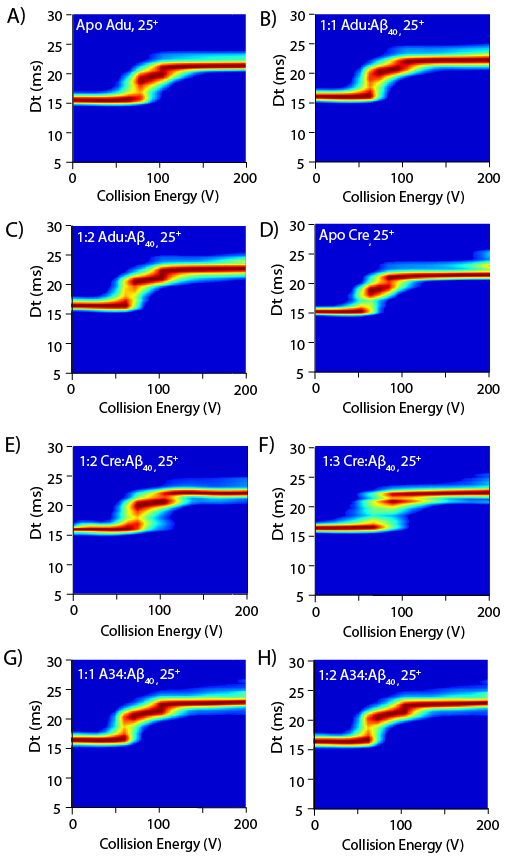


Figure S3 CIU fingerprint of full length mAbs and the mAbs:Aꞵ40 complexes at 25+ charge state: A) Apo Adu B) 1:1 Adu:Aꞵ_40_ C) 1:2 Adu:Aꞵ_40_ D) Apo Cre E) 1:2 Cre:Aꞵ_40_ F) 1:2 Cre:Aꞵ_40_ G) 1:1 A34:Aꞵ_40_ H) 1:2 A34:Aꞵ_40_


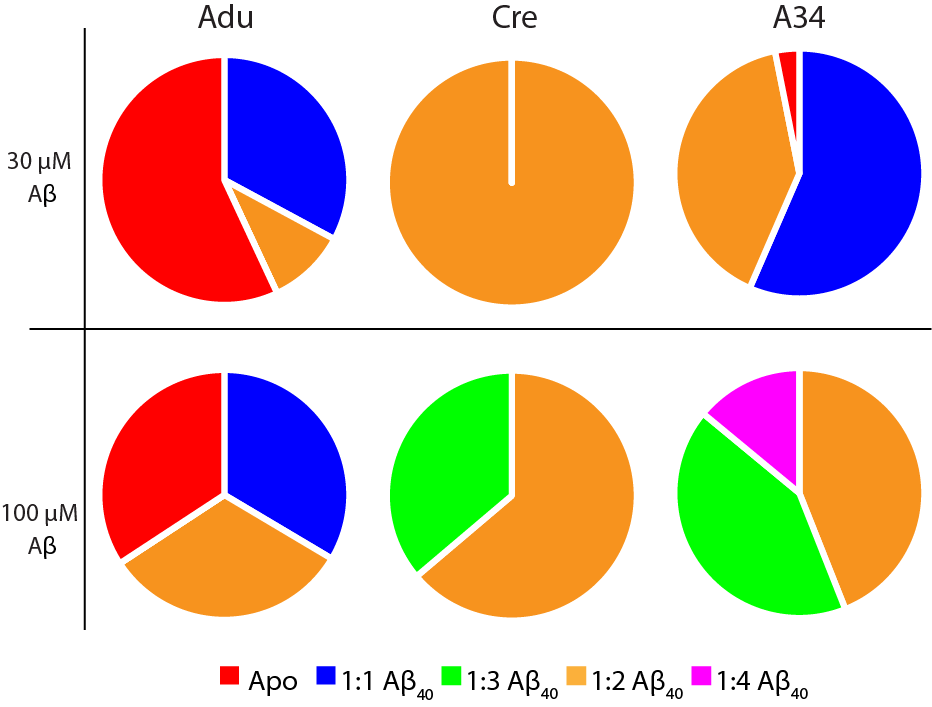


Figure S4 Pie chart depicting the percentage of bound Aꞵ40 for Adu, Cre, and A34 at concentrations of 30 μM and 100 μM
